# Mutations in the Testis-Specific Enhancer of *SOX9* in the *SRY* Independent Sex-Determining Mechanism in the Genus *Tokudaia*

**DOI:** 10.1371/journal.pone.0108779

**Published:** 2014-09-29

**Authors:** Ryutaro Kimura, Chie Murata, Yoko Kuroki, Asato Kuroiwa

**Affiliations:** 1 Graduate School of Life Science, Hokkaido University, Sapporo, Hokkaido, Japan; 2 Graduate School of Life Science, Hokkaido University, Sapporo, Hokkaido, Japan; 3 RIKEN, Center for Integrative Medical Sciences, Yokohama, Kanagawa, Japan; 4 Laboratory of Animal Cytogenetics, Department of Biological Sciences, Faculty of Science, Hokkaido University, Sapporo, Hokkaido, Japan; Colorado State University, United States of America

## Abstract

*SRY* (sex-determining region Y) is widely conserved in eutherian mammals as a sex-determining gene located on the Y chromosome. SRY proteins bind to the testis-specific enhancer of *SOX9* (TES) with SF1 to upregulate *SOX9* expression in undifferentiated gonads of XY embryos of humans and mice. The core region within TES, named TESCO, is an important enhancer for mammalian sex determination. We show that TESCO of the genus *Tokudaia* lost enhancer activity caused by mutations in its SRY and SF1 binding sites. Two species of *Tokudaia* do not have the Y chromosome or *SRY*, and one species has multiple *SRY*s located on the neo-Y chromosome consisting of the Y fused with an autosome. The sequence of *Tokudaia* TESCO exhibited more than 83% identity with mouse TESCO, however, nucleotide substitution(s) were found in two out of three SRY binding sites and in five out of six SF1 binding sites. TESCO of all species showed low enhancer activity in cells co-transfected with SRY and SF1, and SOX9 and SF1 in reporter gene assays. Mutated TESCO, in which nucleotide substitutions found in SRY and SF1 binding sites were replaced with mouse sequence, recovered the activity. Furthermore, SRYs of the *SRY*-positive species could not activate the mutated TESCO or mouse TESCO, suggesting that SRYs lost function as a sex-determining gene any more. Our results indicate that the *SRY* dependent sex-determining mechanism was lost in a common ancestor of the genus *Tokudaia* caused by nucleotide substitutions in SRY and SF1 binding sites after emergence of a new sex-determining gene. We present the first evidence for an intermediate stage of the switchover from *SRY* to a new sex-determining gene in the evolution of mammalian sex-determining mechanism.

## Introduction

In most mammals, testis differentiation is triggered by expression of the Y chromosome-linked gene, *SRY*/*Sry* (sex-determining region Y) [1], [2], [3]. The expression starts in supporting cell precursors derived from coelomic epithelium [4], [5] and induces transcription factor *SOX9*/*Sox9* [6]. Transgenic mouse studies have shown the crucial roles of *Sox9* in testis development: ectopic expression in XX gonads induces testis formation [7], whilst loss-of-function of the gene causes XY sex reversal, resulting in failure to form Sertoli cells [8]. Gonadal sex reversals caused by misexpression of *SOX9* have also been reported in human and European roe deer (*Capreolus capreolus*) [9], [10], [11], and this suggests that the roles of the gene in testis differentiation are widely shared in mammals.

*Sox9* expression in early testis development is regulated by an enhancer named TESCO (TES [testis-specific enhancer of *Sox9*] COre), which is located 13 kb upstream of *Sox9* in mouse. SRY directly binds the enhancer together with SF1 (nuclear receptor subfamily 5, group A, member 1; also referred to as NR5A1) and synergistically upregulates the expression of *Sox9* [12]. After the expression reaches its threshold, SOX9 maintains its own expression via TESCO. FOXL2 (forkhead-box L2) also binds the enhancer to suppress *Sox9* expression in developed ovaries [13], and DAX1 (nuclear receptor subfamily 0, group B, member 1; also referred to as NR0B1) and the Wnt/beta-catenin pathway may antagonize activation of TES by reducing binding of SF1 to TES [14], [15]. These results suggest that TESCO is equally important in repressing *Sox9* in the development and/or maintenance of ovaries in mouse.

However, the genus *Tokudaia*, Muridae, Rodentia does not adopt this sex-determining mechanism that is strictly conserved in mammals. *Tokudaia* consists of three species: *Tokudaia muenninki* (the Okinawa spiny rat), *Tokudaia osimensis* (the Amami spiny rat), and *Tokudaia tokunoshimensis* (the Tokunoshima spiny rat). All inhabit the southernmost islands in Japan: Okinawa-jima Island, Amami-oshima Island, and Tokunoshima Island, respectively. Remarkably, *T. osimensis* and *T. tokunoshimensis* both have XO/XO sex chromosome constitution and lack the Y chromosome. The chromosome numbers of *T. osimensis* and *T. tokunoshimensis* are 2*n* = 25 and 2*n* = 45, respectively [16], [17]. Not only do they lack the Y chromosome, but *SRY* does not exist in their genome [18], [19], which means that they have acquired a unique sex-determining mechanism independent of *SRY*. Two or three additional copies of *CBX2* (chromobox protein homolog 2, also referred to as *M33*), known to be involved in the regulation of *SRY*/*Sry* [20], [21], are present in males of these species [22]. Although it is speculated that this gene might be involved in testis determination through gene dosage effects, no direct evidence or clear mechanisms have been shown.

By contrast, the chromosome number of *T. muenninki* is 2*n* = 44 with XX/XY sex chromosome constitution [23], [24]. Their sex chromosomes are enlarged due to a fusion of a pair of autosomes to the X and Y chromosomes [25]. Remarkably, *T. muenninki* possesses at least 24 haplotypes of the *SRY* gene on the Y chromosome, only three of which contain a full-length ORF (open reading frame) [24]. However, within all of the three copies, the amino acid at the 21st position in the HMG-box is replaced by serine from alanine, but is conserved as alanine in SRY of other eutherian mammals and even in the HMG-box of other members of the SOX-family, including SOX3, SOX8, SOX9, and SOX10. Because it resides in a DNA-binding surface site in the HMG-box, it is suggested that the transcriptional ability of all SRY proteins produced in this species is weakened by this substitution.

In this study, we determined the sequences of TESCO in the three *Tokudaia* species, and performed *in vitro* reporter gene assays to examine the activities of TESCO in these species. The reporter gene assay showed that both SRY and SOX9 failed to activate TESCO of all species, including XO species without *SRY* and also XY species with *SRY*, indicating that TESCO lost enhancer function in the genus *Tokudaia*. Moreover, we determined that substitution of the ancestral *SRY* gene into *T. muenninki* does not restore transcriptional activity.

## Results

### TESCO of *Tokudaia* species was conserved, other than in binding sites

**Figure 1:**
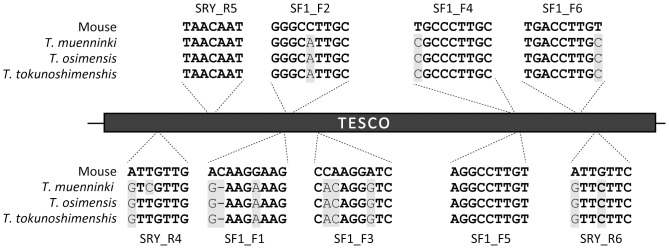
Nucleotide substitutions in the SRY and SF1 binding sites of *Tokudaia* TESCO. Identical residues are indicated by boldface. *Tokudaia* species-specific nucleotides are indicated by gray boxes. The number of each binding site is in accordance with a previous study [12].

To isolate the enhancer in three species of *Tokudaia*, a primer set was designed in a highly conserved region of the mouse and rat TESCO. The sequence of PCR products exhibited more than 94% identity within the genus and 83% identity with mouse TESCO (Table 1). The binding sites of SRY and SF1 within TESCO have been previously reported in mouse [12]. Within *Tokudaia* TESCO, however, nucleotide substitution(s) were found in two out of three SRY binding sites (R4 and R6) and in five out of six SF1 binding sites (F1, F2, F3, F4, and F6) (Fig. 1, Fig. S1) [12]. A few SNPs were also found in TESCO among individuals, but not in the binding sites.

**Table 1:** The identity of TESCO sequences.

	Identity (%)			
Species (bp)	*T. muenninki*	*T. osimensis*	*T. tokunoshimensis*	Mouse
*T. muenninki* (1,363)	-	94.9	94.6	84.2
*T. osimensis* (1,358)	-	-	95.1	83.2
*T. tokunoshimensis* (1,363)	-	-	-	84.2
Mouse (1,292)	-	-	-	-

We then obtained BAC clones containing TESCO by PCR screening of BAC libraries derived from a male *T. muenninki* (XY) and a male *T. osimensis* (XO). Each BAC clone of *T. muenninki* and *T. osimensis* was mapped to the short arms of the X and Y chromosomes (Fig. S2A, B) and 3q23 (Fig. S2C, D), respectively. These locations corresponded with the *SOX9* locations previously mapped in each species [24], [22]. To estimate the length from *SOX9* to TESCO, forward and reverse primers were designed in TESCO and *SOX9*. The PCR product amplified using the *T. osimensis* BAC clone was around 13 kb (Fig. S2E). This size was approximately identical with that of mouse (−12575 bp).

The sequence identities of *SOX9* and *SF1* between mouse and *T. muenninki* homolog are shown in Table S1. One amino acid substitution was found in *T. muenninki* SF1. However, we assumed this substitution did not affect its function, and mouse SF1 sequence was used in all experiments.

### TESCO of *Tokudaia* has no enhancer activities

To examine whether TESCO of *Tokudaia* can be activated by SRY/SOX9 and SF1 *in vitro*, we performed a reporter gene assay using COS7 cells. The ORFs of the *T. muenninki* homologs of *SRY2* [24] and *SOX9* were cloned into expression vectors (named TMU_SRY and TMU_SOX9, respectively), as were mouse *Sry* and *Sox9* (mSRY and mSOX9, respectively). The luciferase vectors containing the promoter of mouse *Sox9* [26], and each of TESCO and the various combinations of expression vectors, were transiently co-transfected into COS7 cells.

**Figure 2:**
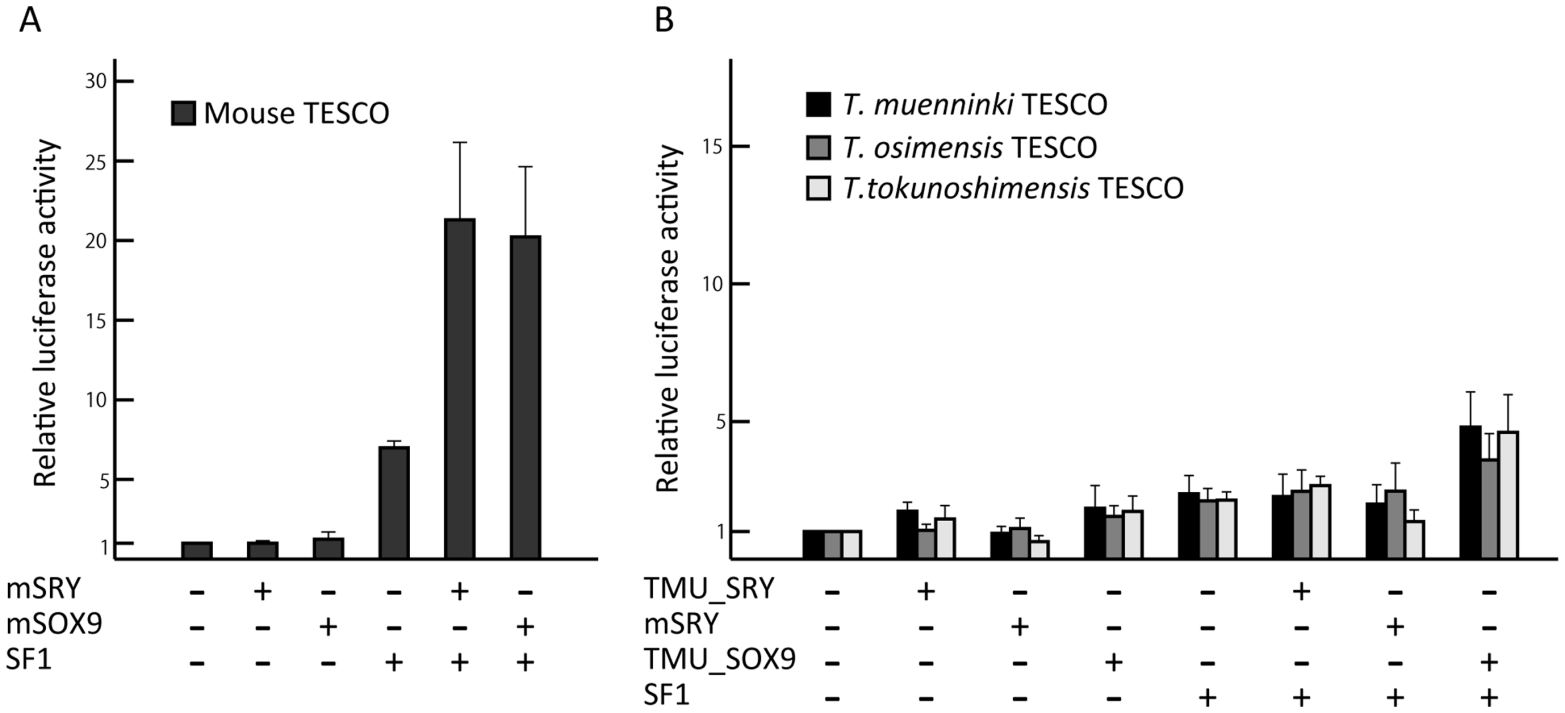
Contrasting enhancer activities in mouse and *Tokudaia* TESCO. (A) Enhancer activities of mouse TESCO performed by reporter gene assays with mSRY or mSOX9 in COS7 cells. The 18–20-fold activation was observed when co-transfected with a combination of SF1 and mSRY or mSOX9. This synergistic regulation of *Sox9* expression corresponded with a previous study [12]. (B) *Tokudaia* TESCO had no enhancer activity. For all *Tokudaia* species and all combinations of transfections, TESCO did not show significant activities. Means and standard deviations from at least three independent experiments are shown.

SF1 by itself stimulated a 7-fold increase in mouse TESCO activity compared to the activity observed when only empty expression vectors were added, whereas mouse TESCO was not significantly activated by mSRY or mSOX9 alone (Fig. 2A). Mouse TESCO showed an approximately 20-fold increase in activity when co-transfected with a combination of SF1 and mSRY or mSOX9, reflecting the synergistic regulation of *Sox9* expression by SRY, SOX9, and SF1. These results corresponded with a previous study [12].

Unlike mouse TESCO, *Tokudaia* TESCO did not exhibit significant activity using any combination of the expressing constructs (Fig. 2B). The SF1-mediated activities of TESCO in the three species were limited to about a 2-fold increase, and the combination of SF1 and TMU_SRY failed to activate any of the *Tokudaia* TESCO, resulting in 2- to 2.5-fold increases in activities. Low activity was also observed in the reporter gene assay using TES in the same manner (Fig. S3).

Since we could not rule out the possibility that the *T. mueninnki* homolog of SRY (TMU_SRY) does not function as a transcription factor, we also carried out the assay using an mSRY. There was not a significant increase in *Tokudaia* TESCO activity (Fig. 2B). After transfecting SF1 and TMU_SOX9-expressing constructs, TESCO showed a limited increase of activities of around 3.5- to 4.7-fold.

### Enhancer activity of *Tokudaia* TESCO was recovered by mutations in binding sites

**Figure 3:**
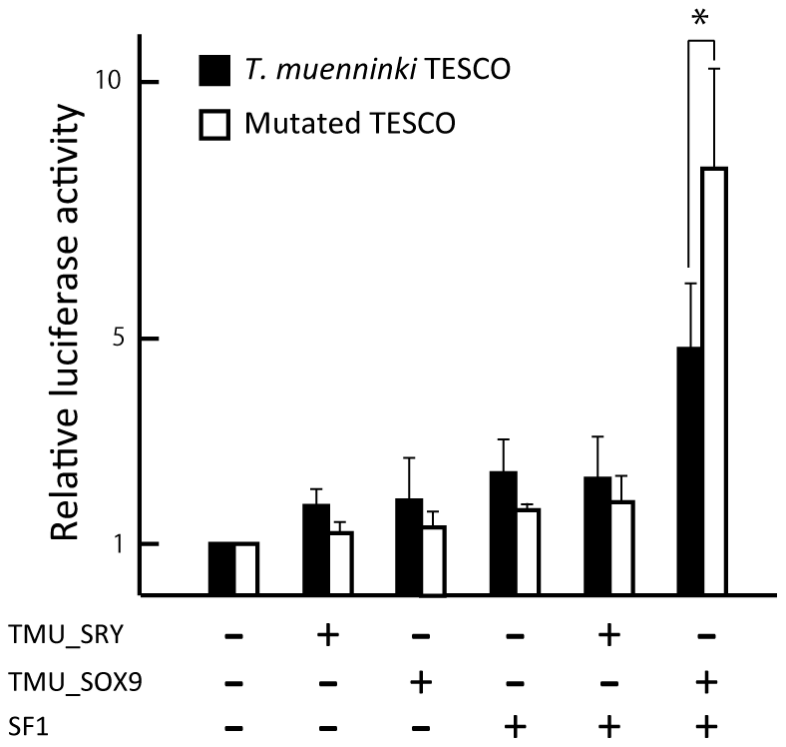
Mutations in the binding sites depress the enhancer activities of *Tokudaia* TESCO. Mutated TESCO, in which all binding sites were replaced by mouse sequence, showed recovery of activities by co-transfection of TMU_SOX9 and SF1. However, no significant activity was observed by TMU_SRY. Means and standard deviations from at least three independent experiments are shown. *P<0.05.

To examine the influences of nucleotide substitutions within the binding sites of SRY and SF1 found in the TESCO of *Tokudaia* (Fig. 1A), all binding sites of *T. muenninki* TESCO were replaced with mouse sequences. A remarkable recovery of the activity was observed when the mutated TESCO was co-transfected with SF1 and TMU_SOX9 (Fig. 3). However, a significant increase of SF1 and TMU_SRY-mediated activation was not confirmed.

### *T. muenninki* SRY had no function as a transcription factor for the upregulation of SOX9

**Figure 4:**
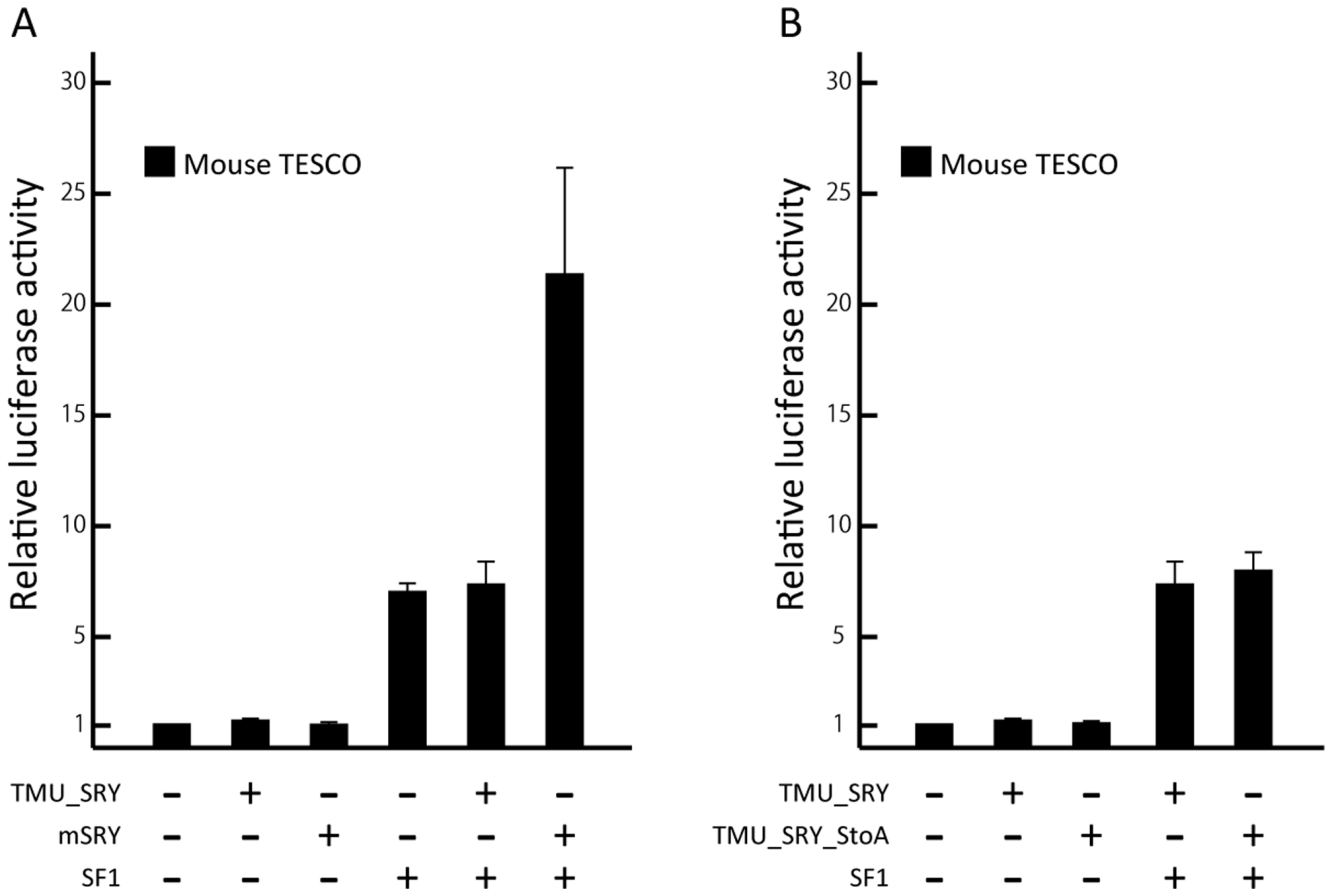
Impaired transcription factor function of *T. muenninki* SRY. (A) Comparison of the enhancer activity of mouse TESCO by co-transfecting mSRY with SF1 and TMU_SRY with SF1. *T. muenninki* SRY did not increase the activity of mouse TESCO. (B) One amino acid substitution at the 21st position within HMG-box had no effect on the function of *T. muenninki* SRY. The TMU_SRY_StoA also did not increase the activity of mouse TESCO. Means and standard deviations from at least three independent experiments are shown.

A previous study reported that activation of mouse TESCO is mediated by human SRY *in vitro* [27]. We therefore examined activation of mouse TESCO mediated by TMU_SRY. However, TMU_SRY-mediated mouse TESCO activation was not observed (Fig. 4A).

In *T. muenninki* SRY, the 21st position in the HMG-box is replaced by serine from alanine [24]. Therefore, we hypothesized that the impaired activation of *T. muenninki* SRY is attributed to this amino acid substitution in the HMG-box. To assess this possibility, we generated a mutated *T. muenninki* SRY construct, whose species-specific alanine was replaced by a serine at the 21st position (TMU_SRY_StoA). TMU_SRY_StoA was co-transfected with mouse TESCO and SF1, resulting in a comparable level of co-transfection with TMU_SRY as shown in Figure 4B.

## Discussion

**Figure 5:**
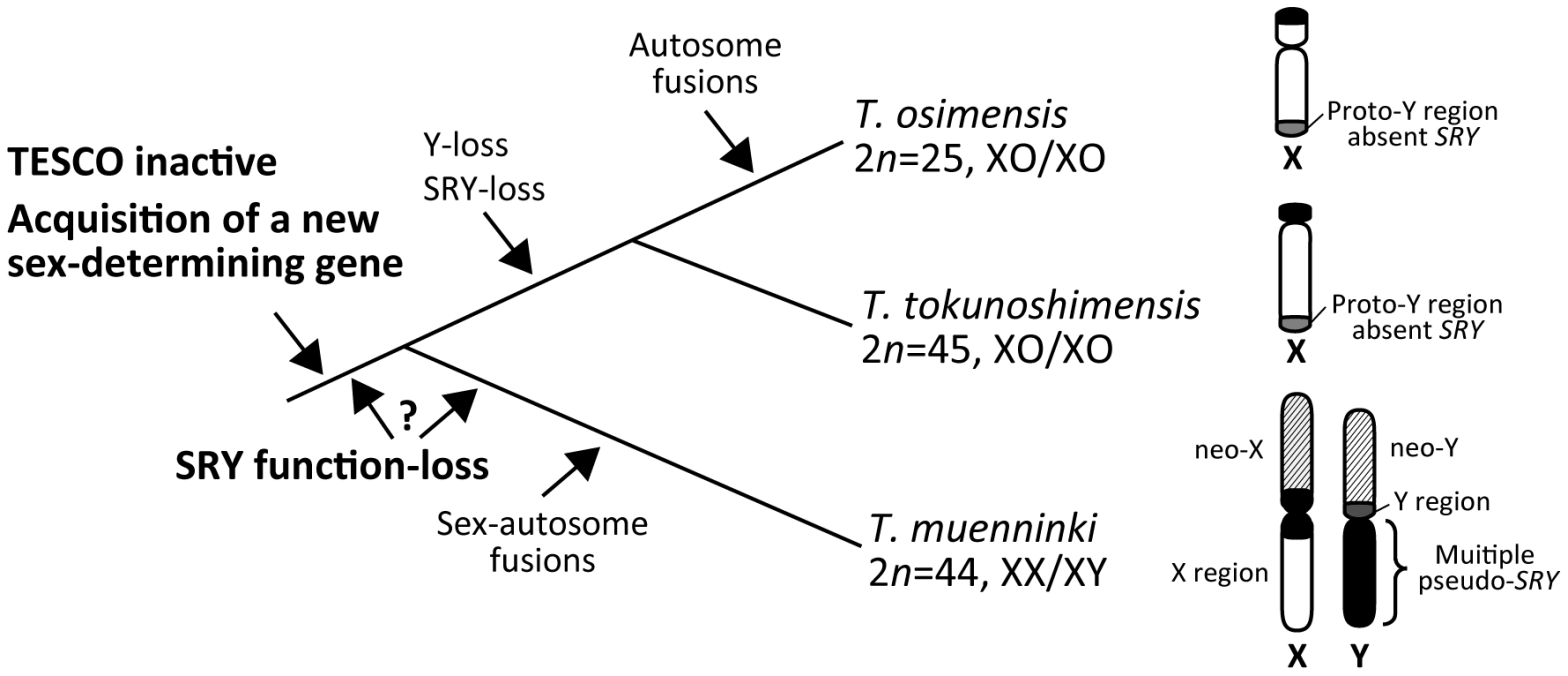
Evolution of the Y chromosome and sex-determining mechanism in the genus *Tokudaia*. Nucleotide substitutions accumulated in SRY and SF1 binding sites in TESCO of the common ancestor after emergence of a new sex-determining gene, leading to inactivation of TESCO, and SRY could not function as a transcription factor. In the common ancestor or in a lineage of *T. muenninki*, SRY itself lost function caused by mutations in the *SRY* sequence. Neo-X and neo-Y chromosomes were acquired by fusion of a pair of autosomes with the X and Y chromosomes. Many pseudo SRY copies amplified and were distributed throughout the heterochromatic region in the neo-Y chromosome. In a common ancestor of *T. osimensis* and *T. tokunosimensis*, a part of the Y region translocated to the distal region of X chromosome and the remaining Y chromosome region containing *SRY* (and other genes) was completely lost.

From our BAC clone analysis, we confirmed that the DNA fragment amplified by PCR was TESCO located upstream of *SOX9*. Despite the relatively high sequence identity with mouse, TESCO of the three *Tokudaia* species was not activated by SRY of mouse or *T. muenninki*. These results indicate that the function of the enhancer to upregulate *SOX9* has been lost not only in *SRY*-negative *T. osimensis* and *T. tokunoshimensis*, but also in SRY-positive *T. muenninki*. We speculated that this functional loss of the enhancer is due to the nucleotide substitutions found within the binding sites of SRY and SF1 in TESCO. Indeed, by replacing the binding sites with those of mouse, TESCO activity in *T. muenninki* mediated by SF1 and TMU_SOX9 was significantly rescued. These substitutions within the binding sites of SRY and SF1 were common to all three *Tokudaia* species, indicating that a loss of TESCO enhancer activity occurred in a common ancestor of *Tokudaia* (Fig. 5). However, TMU_SRY could not activate even the mutated TESCO.

Although *T. muenninki* is the only species in the genus *Tokudaia* that has the *SRY* gene, it is not clear whether the gene retains the transcriptional activity for testis determination. Transgenic expression of human SRY or goat SRY can cause sex reversal in XX mice [28], [29], and activation of mouse TESCO can be mediated by human SRY *in vitro* [27]. Thus, if *T. muenninki* SRY has transcriptional activity, the gene should be capable of activating mouse TESCO in the reporter gene assay. However, in our assay, *T. muenninki* SRY failed to activate mouse TESCO, suggesting that SRY lost its function. We previously reported that the 21st position in the HMG-box of *T. muenninki* SRY is serine, but it is highly conserved as alanine in most mammals [24]. We tried a reporter gene assay using mutated SRY where a species-specific serine was replaced by a conserved alanine at the 21st position. However, no significant increase of transcriptional activity was observed. Therefore, we hypothesized that the impaired activation of *T. muenninki* SRY was caused by variations in the sequence other than the substitution at the 21st position. All *Tokudaia* species are on the verge of extinction and successful artificial breeding has not been reported, so it is virtually impossible to obtain tissues from an embryo of *T. muenninki* to detect endogenous expression of *SRY* or to perform *in vivo* experiments. To evaluate the functionality, further analysis is needed, such as *in vitro* and *ex vivo* experiments using mouse gonads.

Our results suggest that the *T. muenninki SRY*s have already become pseudogenes and a new sex-determining gene superseding *SRY* has been acquired in the two species that lack *SRY*. *T. muenninki* may be in an intermediate stage of the switchover to a new sex-determining gene that has never been observed in mammals. *T. muenninki* has the neo-X and neo-Y regions acquired by fusions of the X and Y chromosomes with a pair of autosomes [25]. A new sex-determining gene may have evolved in the neo-Y region.

The SOX9-mediated activations of *Tokudaia* TESCO were also limited compared with the mouse counterpart. In a previous study, copy number variants of a long distance regulatory region named *RevSex*, located 517–595 kb upstream of *SOX9*, were discovered in human DSD (disorders of sex development) patients and seem to be associated with gonadal expression of *SOX9* [30]. Duplications of this region upstream of *SOX9* cause XX DSD in the absence of *SRY*, and the deletion causes XY DSD, implying the existence of other enhancers that work in concordance with a testis-specific enhancer such as TESCO and/or other regulatory elements for the gonad-specific expression pattern of *SOX9* [30], [31]. In addition, a promoter region inserted with a transgene 0.98 Mb upstream of the *Sox9* gene drives *Sox9* expression and induces XX sex reversals in *Odd Sex* mice [32]. These facts shed light on the possibility that, in *Tokudaia* species, the *SOX9* expression in bipolar gonads in males is driven by enhancers other than TESCO.

Although TESCO sequence is conserved only in eutherian mammals, an evolutionarily conserved region (ECR) of approximately 180 bp within TESCO is present in a variety of vertebrates including marsupials, monotremes, birds, reptiles, and amphibians [33]. It is a common regulatory system of *SOX9*/*Sox9* expression in gonads across a wide range of vertebrates. ECRs were also highly conserved in the three species of *Tokudaia* (Fig. S1). *SOX9* has multiple functions other than testis differentiation: expression is noted at sites of chondrogenesis in mouse embryo [34], and heterozygous mutation is associated with the bone dysmorphology syndrome, campomelic dysplasia (CD) [9], [35]. The ECR may be widely used for *SOX9* upregulation in vertebrate species not associated with SRY.

Based on these observations, we propose a switching process of the sex-determining gene in the genus *Tokudaia* (Fig. 5). Our molecular phylogenic analysis reveals that *T. muenninki* diverged earliest from the common ancestor of three species [24]. Nucleotide substitutions accumulated in SRY and SF1 binding sites in TESCO of the common ancestor after emergence of a new sex-determining gene. As a result, TESCO became inactive and SRY could not function as a transcription factor. In the common ancestor or in a lineage of *T. muenninki*, SRY itself lost function due to mutations in the *SRY* sequence. Chromosome fusion of a pair of autosomes with the X and Y chromosome occurred, and many pseudo SRY copies amplified and were distributed throughout the heterochromatic region. In a common ancestor of *T. osimensis* and *T. tokunosimensis*, a part of the Y region translocated to the distal region of the X chromosome and the remaining Y chromosome region containing *SRY* (and other genes) was completely lost.

We showed evidence for an intermediate stage of the switchover from *SRY* to a new sex-determining gene in eutherian species. Future research should focus on finding a new sex-determining gene, other than *SRY*, which has never been reported in mammals.

## Materials and Methods

### Animals

The three species of *Tokudaia* are all endangered (The IUCN Red List of Threatened Species; http://www.iucnredlist.org/10/5/2014) and have been protected by the Japanese government as natural treasures since 1972. With permission from the Agency for Cultural Affairs and the Ministry of the Environment in Japan, we captured *T. muenninki* on Okinawa-jima Island in March 2008 and February 2009 [36], *T. osimensis* on Amami-Oshima Island in February 2004, and *T. tokunoshikensis* on Tokunoshima Island in March 2005. The tips of their tails were cut by surgical scissors to obtain DNA and fibroblasts for cell culture. Tissue samples of brains and testes were obtained from animals that died in accidents or naturally. All the animal experiments in this study were approved by Institutional Animal Care and Use Committee of National University Corporation Hokkaido University and performed in accordance with the Guidelines for the Care and Use of Laboratory Animals, Hokkaido University.

### Cloning and sequencing of TESCO

We designed a PCR primer set for amplification of TES by comparing the sequences of mouse and rat [12]. The primer sequence is shown in Table S2. Genomic DNA extracted from one male of each species was used as a template for PCR. The accession numbers of TES of each species are as follows: *T. muenninki*, AB916964; *T. osimensis*, AB916967; and *T. tokunoshimensis*, AB916971. We used TESCO sequence within TES for reporter gene assays. To identify the nucleotide polymorphism, we determined the TESCO sequence in three males and three females in each species. The primer sequence is shown in Table S2. The accession numbers of TESCO of each species are as follows: *T. muenninki*, AB916965 and AB916966; *T. osimensis* AB916968 and AB916969; and *T. tokunshimensis*, AB916972.

### Construction of BAC libraries and Isolation of BAC clones

BAC libraries were constructed according to the procedures previously described [37]. Cultured fibroblast cells were embedded in 1% agarose gel, treated with *Sac I*, and subjected to pulsed-field gel electrophoresis. The DNA fragments ranging from 125 to 225 kb were isolated and ligated with pKS145 vector. Transformation was carried out electronically using *E. coli* DH10B as a host strain. Ampicillin-resistant transformants were collected and stored in 384-format plates. A PCR primer set was designed and used to screen the *T. muenninki* and *T. osimensis* BAC libraries using a two-step 3D PCR screening system [37]. Identity of positive clones was confirmed by PCR using a single colony as the template DNA. The experimental number of obtained BAC clone of *T. muenninki* and *T. osimensis* are TMB1-199P19 and TOB1-211A22, respectively.

### Estimation of the length from SOX9 to TESCO

A PCR primer set was designed to estimate the length from SOX9 to TESCO. Forward and reverse primers were designed in TESCO and *SOX9* sequences, respectively (Table S2).

### Preparation of chromosomes and FISH mapping

The preparation of R-banded chromosomes and FISH was performed as previously described [38], [39]. Fibroblasts obtained from tails of male *T. muenninki* and *T. osimensis* were cultured in Chang Medium (Irvine Scientific) and/or DMEM supplemented with 12% fetal bovine serum at 37°C in an atmosphere of 5% CO_2_. BAC clones of *T. muenninki* and *T. osimensis* containing *SOX9* and TESCO were used as probes. The BAC clones were labeled by nick translation with biotin-16-dUTP (Roche Diagnostics) according to the manufacturer's protocol. A mixture (20 µl) containing the labeled DNA was placed on the denatured chromosome slides and covered with parafilm, and the slides were incubated overnight at 37°C. After being washed in 4× SSC, the slides were incubated under parafilm for 1 h with fluoresceinated avidin (Roche) at 1∶500 dilution in 1% BSA/4× SSC. The slides were washed on a shaker with 4× SSC, 0.01% Nonidet P-40y in 4× SSC, and 4× SSC, each for 10 min. The chromosome slides were stained with propidium iodide (0.75 ug/ml).

### Cloning of *SOX9* and *SF1* cDNA of *T. osimensis*

Total RNA was extracted from the brain of an adult female and adult testis of *T. osimensis* using RNeasy Mini Kit (QIAGEN) according to the manufacturer's instructions. The total RNA was reverse transcribed using SuperScript III (Invitrogen) and oligo(dT) primers. We designed two sets of degenerate PCR primers by comparing the *Sox9*/*SOX9* sequences, including the 5′-UTR and 3′-UTR of mouse (NM_011448) and rat (XM_003750950). A sequence with a complete ORF was determined by combining partial sequences of *SOX9* amplified by each degenerate PCR primer set. After determining partial sequences, a primer set was also designed to cover a complete ORF of *SOX9*. The sequence of each primer is shown in Table S2. The accession number of the *T. osimensis* homolog of *SOX9* is AB911120. The *T. osimensis* homolog of *SF1* was obtained by the same method. The sequence of each primer is shown in Table S2. The accession number of the *T. osimensis* homolog of *SF1* is AB911121.

### Construction of plasmids for enhancer analysis

pcDNA3.1 (+) (Invitrogen) was used as an expression vector. A complete ORF of *T. muenninki SRY* was cloned as previously described [24]. We used *SRY2* (AB548700) cloned into a plasmid as a template. A forward primer and a reverse primer with *BamH*I and *Xho*I restriction sites, respectively (Table S2), were designed to amplify the *SRY* ORF. The PCR product was cloned into the *BamH*I/*Xho*I sites of the expression vector. The mutated *SRY*, which has an amino acid substitution from a species-specific alanine to a serine at the 24th position of *T. muenninki* SRY, was generated by introducing a site-specific mutation into *T. muenninki SRY* using gene splicing by overlap extension PCR [40]. Briefly, a set of complementary primers containing a nucleotide substitution was designed. The first and second PCR were performed using this primer set and the primer set described in the cloning of *T. muenninki SRY*. The *BamH*I/*Xho*I fragments derived from *SOX9* and *SF1* cDNA clones were inserted into the expression vectors. The *Sox9* promoter (−204 to +327) [41] was PCR-amplified using mouse genomic DNA with a forward and reverse primer with *Bgl*II and *Hind*III restriction sites, respectively. The product was ligated into a *Hind*III site of the pGL3-Basic vector (Promega). Subsequently, each TESCO of three species of *Tokudaia* and mouse (as a positive control) was amplified with primer sets with *Kpn*I and *Bgl*II restriction sites (Table S2), and ligated in the pGL3 containing the mouse *Sox9* promoter. To generate a mutated *T. muenninki* TESCO reporter construct that has the same binding sites of SF1 and SRY with mouse, site-specific mutations were made in *T. muenninki* TESCO using gene splicing by overlap extension PCR, as described in the construction of mutated SRY. The sequence of each primer is shown in Table S2.

### Reporter gene assays

COS7 cells were cultured in DMEM supplemented with 10% fetal bovine serum at 37°C in an atmosphere of 5% CO_2_. COS7 cells were seeded at a density of 0.5×10^5^ per well 24 h prior to transfection. Transfection was conducted using 2 ul of Lipofectamine 2000 (Invitrogen). Either 550 ng of TESCO-Sox9 promoter-luc construct or 550 ng of Sox9 promoter-luc construct, several combinations of 110 ng of each expression vector, and 30 ng of Renilla luciferase control reporter vectors, pRL (Promega), were transfected according to the manufacturer's instructions. The total amount of expression vector was adjusted to 220 ng by empty pcDNA. Forty-eight hours after the transfection, the reporter activities were measured by Dual-Luciferase Reporter Assay System (Promega) in accordance with the manufacturer's instructions. The reporter activity was normalized to Renilla luciferase activity as an internal control. Each experiment was carried out in triplicate.

## Supporting Information

Figure S1 **Comparison of TESCO sequences among mouse and three** ***Tokudaia*** **species.** Black and gray boxes highlight three SRY binding sites (R4, R5, and R6) and six SF1 binding sites (F1, F2, F3, F4, F5, and F6) shown in a previous study [12], respectively. Closed gray boxes highlight five ECRs shown in a previous study [33].
(TIF)

Figure S2 **Chromosome location of the BAC clones containing TESCO in** ***Tokudaia*** **species.** The BAC clones were mapped to the short arms of the X and Y chromosomes in *T. muenninki* (A, B) and 3q23 in *T. osimensis* (C, D), respectively. Propidium iodide-stained R- (A, C) and Hoechst G-banding patterns (B, D) are demonstrated in the right and left panels, respectively. Scale bar indicates 10 um. Estimation of the length from *SOX9* to TESCO (D). The PCR products amplified a primer set spanning *SOX9* and TESCO that was approximately 17 kb.
(TIF)

Figure S3 **Enhancer activities in mouse and** ***T. muenninki*** **TES.** Enhancer activities of mouse and *T. muenninki* TES performed by reporter gene assays with mSRY, TMU_SRY or TMU_SOX9 in COS7 cells. *T. muenninki* TES showed low enhancer activity in all combinations of co-transfection. Means and standard deviations from at least three independent experiments are shown. *P<0.05; **P<0.1.
(TIF)

Table S1 **The identity of SOX9 and SF1 sequences between mouse and** ***Tokudaia*** **.**
(XLSX)

Table S2 **Primer list.**
(XLSX)
