# Supplementary figures and images for: Mutations in the Testis-Specific Enhancer of SOX9 in the SRY Independent Sex-Determining Mechanism in the Genus Tokudaia
Source: PLoS One. 2014 Sep 29;9(9):e108779. doi: 10.1371/journal.pone.0108779 (PMC4181316; doi:10.1371/journal.pone.0108779)

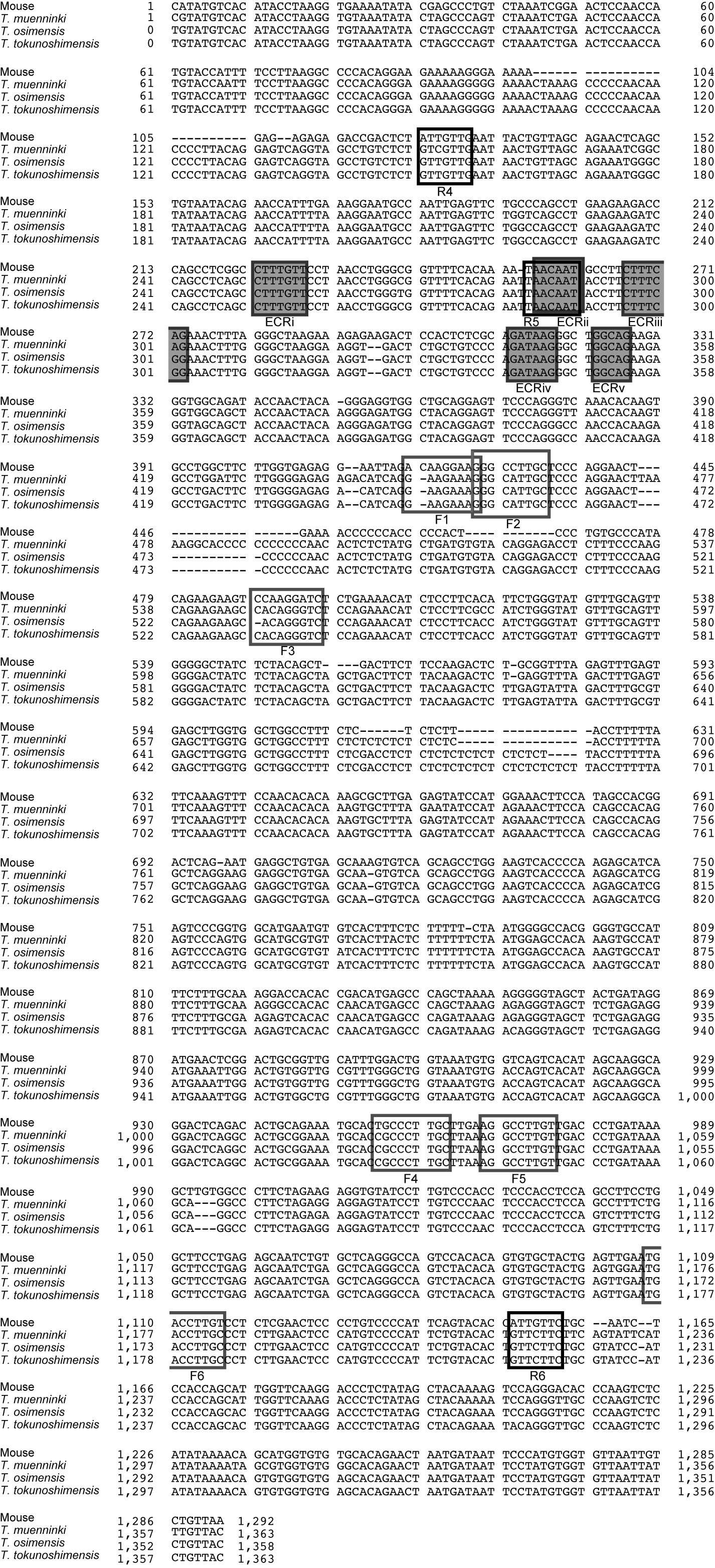

Supplement: Figure S1 — Comparison of TESCO sequences among mouse and three Tokudaia species. Black and gray boxes highlight three SRY binding sites (R4, R5, and R6) and six SF1 binding sites (F1, F2, F3, F4, F5, and F6) shown in a previous study [12], respectively. Closed gray boxes highlight five ECRs shown in a previous study [33]. (TIF) [file pone.0108779.s001.tif]

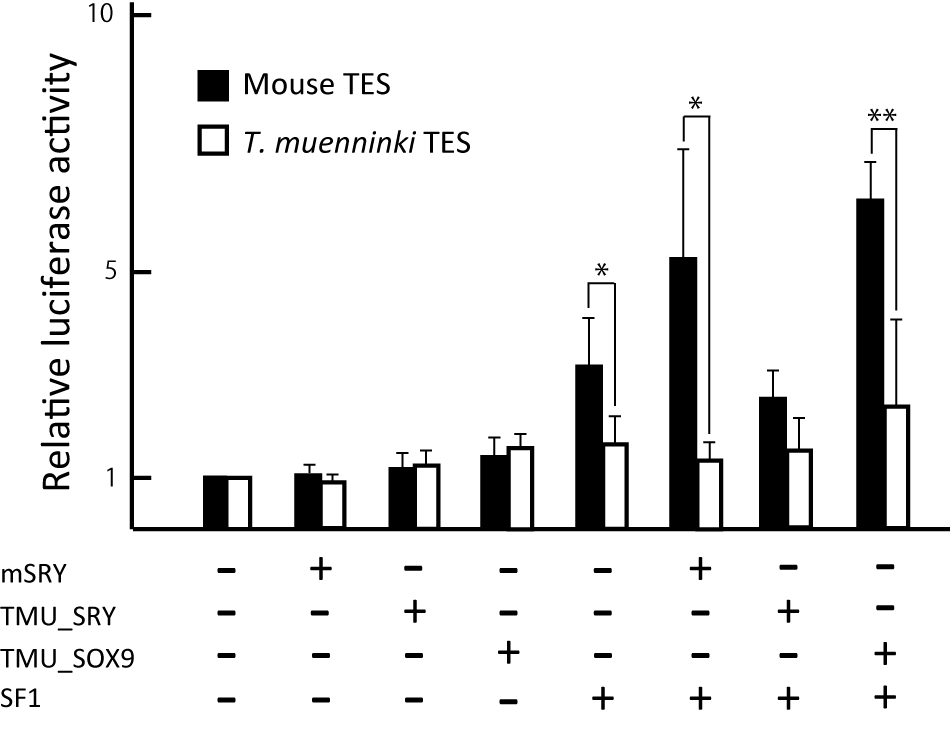

Supplement: Figure S3 — Enhancer activities in mouse and T. muenninki TES. Enhancer activities of mouse and T. muenninki TES performed by reporter gene assays with mSRY, TMU_SRY or TMU_SOX9 in COS7 cells. T. muenninki TES showed low enhancer activity in all combinations of co-transfection. Means and standard deviations from at least three independent experiments are shown. *P<0.05; **P<0.1. (TIF) [file pone.0108779.s003.tif]
